# An Adjuvanted, Tetravalent Dengue Virus Purified Inactivated Vaccine Candidate Induces Long-Lasting and Protective Antibody Responses Against Dengue Challenge in Rhesus Macaques

**DOI:** 10.4269/ajtmh.14-0268

**Published:** 2015-04-01

**Authors:** Stefan Fernandez, Stephen J. Thomas, Rafael De La Barrera, Rawiwan Im-erbsin, Richard G. Jarman, Benoît Baras, Jean-François Toussaint, Sally Mossman, Bruce L. Innis, Alexander Schmidt, Marie-Pierre Malice, Pascale Festraets, Lucile Warter, J. Robert Putnak, Kenneth H. Eckels

**Affiliations:** US Army Medical Component–Armed Forces Research Institute of Medical Sciences (USAMC-AFRIMS), Bangkok, Thailand; Walter Reed Army Institute of Research (WRAIR), Silver Spring, Maryland; GlaxoSmithKline Vaccines, Rixensart, Belgium; GlaxoSmithKline Vaccines, King of Prussia, Pennsylvania

## Abstract

The immunogenicity and protective efficacy of a candidate tetravalent dengue virus purified inactivated vaccine (TDENV PIV) formulated with alum or an Adjuvant System (AS01, AS03 tested at three different dose levels, or AS04) was evaluated in a 0, 1-month vaccination schedule in rhesus macaques. One month after dose 2, all adjuvanted formulations elicited robust and persisting neutralizing antibody titers against all four dengue virus serotypes. Most of the formulations tested prevented viremia after challenge, with the dengue serotype 1 and 2 virus strains administered at 40 and 32 weeks post-dose 2, respectively. This study shows that inactivated dengue vaccines, when formulated with alum or an Adjuvant System, are candidates for further development.

## Introduction

The dengue viruses (DENVs) are enveloped, single-stranded RNA viruses in the family *Flaviviridae*.^1^,^2^ There are four antigenically related DENV serotypes (serotypes 1–4) that can be distinguished serologically by virus neutralization tests. The message-sense genome contains a single open-reading frame encoding three structural proteins (capsid, membrane [M] and its precursor pre-membrane [prM; prM/M]), and envelope [E]) and seven non-structural (NS) proteins.^3^ The virion contains 180 copies of E protein, 180 copies of M and/or prM, and a nucleocapsid core.^4^

An estimated 390 million dengue infections occur each year, of which 96 million are estimated to be clinically apparent.^5^ After infection, there seems to be long-term immunity to the infecting serotype but only transient immunity to heterologous serotypes.^6^ Neutralizing antibodies (NAbs) directed against epitopes on the virion E antigen are believed to contribute to protection against clinical disease.^7^ In addition to anti-E antibodies, other antibodies (e.g., anti-NS1 and anti-prM/M) and cell-mediated immune (CMI) responses might play roles in both immunoprotective as well as immunopathogenic profiles.^8^–^10^ Although primary dengue infection, especially in young children, is often mildly symptomatic or even asymptomatic,^11^ dengue-primed individuals seem to be at greater risk for developing severe disease after a second, heterologous dengue infection characterized by severe plasma leakage, hemorrhage, and/or end-organ dysfunction after defervescence.^12^,^13^

The immunologic mechanisms determining infection severity are largely unknown, and several hypotheses have been advanced. One widely accepted hypothesis, known as antibody-dependent enhancement (ADE), states that, during a secondary DENV infection, DENV serotype cross-reactive antibodies against E,^14^ and possibly prM,^15^ induced by the primary infection can enhance replication of the second (heterologous) infecting DENV serotype in Fc-receptor (FcR) -bearing cells.^16^,^17^ There is also evidence that rapid expansion of cross-reactive T cells can result in dysregulated cytokine expression and up-regulation of FcR expression, possibly leading to enhanced viral replication and vascular leakage.^10^ These unique features of DENV infection emphasize the need for a vaccine that is able to protect against disease caused by each of the four DENV serotypes.

The DENV vaccine candidate farthest advanced in clinical trials is the chimeric yellow fever (YF) 17D/dengue 1–4 (CYD) vaccine (Chimerivax, Sanofi Pasteur, Lyon, France), which contains the YF 17D live-attenuated virus backbone expressing the DENV prM and E genes. However, three vaccine doses spaced over 12 months are required to maximize seroconversion and NAb responses against all four DENV serotypes.^18^,^19^ In a recent phase 2b efficacy trial in children in Thailand, the CYD vaccine proved to be safe and induce NAb; however, the primary endpoint (statistically significant protection against dengue illness) was not reached for all serotypes.^20^ The reason why the vaccine failed to protect against disease after DENV-2 infection remains unclear, particularly because relatively high-titered anti–DENV-2 NAb responses were detected 4 weeks post-dose 3, as tested in a subset of subjects.

Other live-attenuated vaccine (LAV) candidates based on attenuating mutations in a DENV backbone have been generated,^21^–^26^ and some have been tested in clinical trials.^25^,^26^ However, the ability of tetravalent live-attenuated DENV vaccines to induce balanced NAb responses to each of the four serotypes can be affected by pre-existing immunity to DENV and possibly, other flaviviruses in the host. This pre-existing immunity, particularly when induced by previous DENV infection or prior vaccination with a DENV LAV, can result in NAb responses that are biased toward only some of the DENV serotypes.^27^,^28^ Consequently, to achieve seroconversion and potent NAb responses against all four DENV serotypes, most dengue LAVs require multiple doses spaced over 3–12 months.^20^,^29^,^30^ This could result in increased susceptibility to severe disease in the intervening time period between vaccine doses and would seem to make such vaccines less suitable for use in certain populations (e.g., travelers and military) or situations (e.g., epidemic interruption). In contrast, non-replicating vaccines, which are less likely to exhibit interference among the virus serotypes, could potentially reduce the time interval required between primary and booster vaccinations to induce protection more rapidly. An example is the recently licensed Vero cell-derived purified inactivated vaccine (PIV) for Japanese encephalitis, which when administered on a 0, 28-day schedule, induced high-titer and long-lasting NAb responses within 1 month after the second dose.^31^,^32^ Moreover, with inactivated vaccines, reversion to a more pathogenic DENV phenotype and subsequent transmission by the vector cannot occur, and such vaccines may be administered to the elderly, chronically ill individuals, and immunosuppressed individuals with less risk. Recent advancements in the development of qualified cell lines for virus propagation and in vaccine production technology, as well as the availability of innovative adjuvants, further support the case for an inactivated DENV vaccine.

Preclinical evaluation of DENV vaccines has been hampered by lack of good animal disease models. Except for some transgenic mouse models for which the predictive value remains unclear, the non-human primate (NHP) infection model, which uses viremia as a surrogate for disease, is among the most widely accepted animal models for evaluating the humoral immunity induced by candidate DENV vaccines and the protective efficacy of such vaccines.^33^–^35^ In a study in rhesus macaques,^36^ two doses, administered 3 months apart, of a monovalent DENV-2 PIV (5-μg antigen dose) formulated with either aluminum hydroxide (alum) or an Adjuvant System prevented DENV-2 viremia (used as an infection marker/disease surrogate^37^) after challenge. The Adjuvant Systems used in this study were AS05 and AS08 (both currently discontinued) as well as AS04. Although there was evidence for circulating challenge viral RNA (RNAemia) by polymerase chain reaction (PCR) assay in most vaccinated animals, the viral load per day was reduced compared with controls for groups that received formulations with alum-based AS04, AS05, or AS08.^36^ In a subsequent study in rhesus macaques, administration of three doses at monthly intervals of an alum-adjuvanted tetravalent DENV (TDENV) PIV (1 μg per serotype) resulted in NAb responses against each of four DENV serotypes.^38^

This report describes two studies performed to evaluate the immunogenicity and protective efficacy of candidate TDENV PIV vaccine formulations in the rhesus macaque infection model when administered using a short two-dose vaccination schedule. In the first study, the tested vaccine formulations contained different antigen doses adjuvanted with AS01, AS03, AS04 (which are among the Adjuvant Systems that are currently used in various clinical studies), or alum. Based on the results, a subsequent study evaluated formulations containing a single antigen dose (0.5 μg per DENV serotype) adjuvanted with alum, AS01, or one of three different dose levels of AS03. The goal was to identify formulations suitable for clinical evaluation.

## Materials and Methods

### Study design.

Study 1 compared the immunogenicity of TDENV PIV antigens without adjuvants with that of different antigen doses of TDENV PIV formulated with alum, AS01_E_, AS03_A_, or AS04_D_. Forty-four flavivirus-naive rhesus macaques were randomly allocated into 11 groups (*N* = 4 per group), each of which received two intramuscular (IM) injections of 0.5 mL TDENV PIV or saline (control) 30 days apart: three groups received TDENV PIV formulations containing antigen doses of 0.125, 0.5, or 2.0 μg/serotype formulated with alum; six other groups received TDENV PIV with antigen doses of 0.125 or 0.5 μg/serotype formulated with AS01_E_, AS03_A_, or AS04_D_; a tenth group received non-adjuvanted TDENV PIV (2.0 μg/serotype); and a negative control group received phosphate-buffered saline (PBS). Serum samples were obtained before each vaccine administration (days 0 and 30), at 1 and 3 months post-dose 2 (days 60 and 120, respectively) and at the end of the observation period (day 150). Dengue serotype-specific NAb titers were determined.

Study 2 evaluated the immunogenicity over a longer term as well as the protective efficacy of TDENV PIV (0.5 μg/serotype) adjuvanted with AS03_A_, AS03_B_, AS03_C_, AS01_E_, or alum, compared with saline (PBS). Sixty flavivirus-naive rhesus macaques were randomly allocated into six groups (*N* = 10 per group). Five groups received two IM injections of 0.5 mL adjuvanted TDENV PIV 28 days apart, and a sixth group received PBS as a control. Serum samples were obtained before each dose (days 0 and 28) and at 4, 12, 20, 32, 36, 40, and 44 weeks post-dose 2 (days 56, 112, 168, 252, 280, 308, and 336). Five animals from each group were randomly selected to be challenged with DENV-2 strain S16803^39^ (passaged four times in Vero cells) on day 252 or DENV-1 strain Puerto Rico/94 (PR/94^40^; passaged two times in Vero cells) on day 308. Dengue serotype-specific NAb titers were determined from sera obtained at all time points up to and including day 168 (*N* = 10 per group) and on days 252, 280, 308, and 336 (*N* = 5 per group). Serum samples were obtained daily post-challenge for 14 days and tested for the presence of live virus (viremia) by virus amplification in Vero cell culture, and for viral RNA (RNAemia) by real-time reverse transcription- (RT-) PCR assay as an alternative infection marker.

### Animals and husbandry.

All work with animals was conducted in compliance with the Animal Welfare Act and other federal statutes and regulations relating to animals and experiments involving animals, and adhered to the principles stated in the *Guide for the Care and Use of Laboratory Animals* (2011 edition). All procedures were reviewed and approved by an Institutional Animal Care and Use Committee, and were performed in a facility accredited by the Association for Assessment and Accreditation of Laboratory Animal Care, International.

Healthy adult rhesus macaques (*Macaca mulatta*) of Indian origin who were colony-born in captivity at the Department of Veterinary Medicine of the US Army Medical Component–Armed Forces Research Institute of Medical Sciences (USAMC-AFRIMS; Bangkok, Thailand) were used. In study 1, animals (male/female ratio of 30/14) were 5–14 years of age on study initiation and ranged from 5.2 to 13.2 kg. In study 2, animals (male/female ratio of 23/37) were 4–10 years of age on study initiation and ranged from 5.1 to 15.8 kg. Before vaccination, animals that exhibited negative serological test results for simian herpes B, simian immunodeficiency virus, and simian T-cell lymphotropic virus were screened by hemagglutination inhibition assay for antibodies to DENV serotypes 1, 2, 3, or 4 or Japanese encephalitis flaviviruses. Animals with positive results were excluded.

Animals were acclimated for 14 days before the start of the inoculation. They were housed individually in stainless steel cages to facilitate the detection of possible vaccine toxicity but retained in a social environment through contact with other monkeys (i.e., visual, auditory, and [when possible], tactile through double-grid partitions). The cages were situated in an indoor space fitted with double insect screens and had increased air ventilation derived from air handling units. Environmental parameters (temperature and humidity) were recorded daily. Monkeys received a complete commercial diet (083G; The Perfect Companion Group Co. Ltd, Bangkok, Thailand) supplemented with mixed fresh fruit or vegetables. Food was withheld for 6–12 hours before any procedure requiring anesthesia, but drinking water was offered *ad libitum*. Animals were fed after recovery from anesthesia.

### Vaccines.

The preparation of the TDENV PIV antigens has been described previously.^41^,^42^ Briefly, the TDENV PIV was based on a tetravalent formulation of non-attenuated viruses of the strains West Pac 74 (DENV-1), S16803 (DENV-2), CH53489 (DENV-3), and TVP360 (DENV-4), propagated in Vero cells, purified, and inactivated with formalin. The PIV antigens (doses of 0.125, 0.5, or 2.0 μg/serotype) were formulated with or without adjuvants (see Study Design). Adjuvants used were alum (Alhydrogel 2%; Brenntag Biosector, Frederikssund, Denmark; 10.38 mg Al^3+^/mL, after dilution containing 500 μg Al^3+^/0.5-mL vaccine dose) and the Adjuvant Systems AS01_E_, AS03_A,_ AS03_B_, AS03_C_, and AS04_D_,^43^–^46^ supplied by GlaxoSmithKline (GSK) Vaccines (Rixensart, Belgium). AS01_E_ is an Adjuvant System containing 25 μg 3-*O*-desacyl-4′-monophosphoryl lipid A (MPL; GSK Vaccines, Rixensart, Belgium) and 25 μg *Quillaja saponaria* fraction 21 (QS-21; Antigenics Inc., a wholly owned subsidiary of Agenus Inc., Lexington, MA) in a liposome-based formulation. AS03_A_, AS03_B_, and AS03_C_ are Adjuvant Systems containing α-tocopherol and squalene in an oil-in-water emulsion (11.86, 5.93, and 2.97 mg α-tocopherol, respectively). AS04_D_ is an Adjuvant System containing MPL (50 μg MPL) adsorbed on aluminum hydroxide (500 μg Al^3+^). The injected volume of each Adjuvant System was one full human dose (0.5 mL).

### Vaccinations and specimen collection.

Before receiving inoculations, monkeys were anesthetized with ketamine (15 mg/kg). Each 0.5-mL dose of the vaccine or control preparation was administered aseptically by IM inoculation into the upper thigh by a 29-gauge, 0.5-in needle and a 1-mL syringe.

Safety was assessed based on the occurrence of local (injection site) reactogenicity events (redness, swelling, muscle induration, ulceration, abscess, or other abnormalities), which were recorded daily for 3 days post-vaccination, and systemic reactions (changes in appetite, behavior, and/or activities, such as depression, lethargy, dehydration, anorexia, changes in urination and/or defecation, diarrhea, and vomiting), which were recorded daily for 7 days post-vaccination.

Before drawing blood, animals were sedated with ketamine (10 mg/kg). For the viremia and microneutralization (MN) assays, 2 and 5 mL blood, respectively, were drawn using a vacutainer with a 20- to 22-gauge, 1- to 1.5-in needle and 2- or 5-mL serum separator tubes, respectively.

### Viral challenge.

Challenge was done with DENV-1 strain PR/94^40^ and DENV-2 strain S16803^39^ on days 308 and 252, respectively (both low-passage strains; Study Design). In a parallel study testing all four serotypes, DENV-1 and DENV-2 challenge strains were selected based on infectivity (number of days of viremia) in flavivirus-naive NHP (data not shown). For inoculation, 0.5 mL sterile culture medium containing 4–5 log_10_ plaque-forming units (pfu) DENV-1 or DENV-2 was administered subcutaneously by a 23-gauge, 1-in needle and a 3-mL syringe at the disinfected loose skin of the upper arm. Residual challenge virus was returned to the laboratory to verify its potency by plaque titration.

### MN assay.

NAbs to each DENV serotype were detected and measured at the Translational Medicine Branch, Pilot Bioproduction Facility, Walter Reed Army Institute of Research (WRAIR) using an in house-qualified, 96-well, high-throughput enzyme-linked immunosorbent assay (ELISA) -based MN test (MN_50_) performed in Vero cells as previously described.^30^ The virus strains to be neutralized were DENV-1 West Pac 74, DENV-2 S16803, DENV-3 CH53489, and DENV-4 341750. Seropositivity was defined as a titer ≥ 1:10 dilution. This MN test was shown to be specific and sensitive for the detection of anti-DENV NAb.

### Virus amplification and cell culture infectivity dose 50 viremia assay.

Amplification of DENV in viremic sera by inoculation of Vero cell cultures has been described previously.^47^ Briefly, serum samples (0.1 mL) were inoculated onto freshly confluent Vero cell (ATCC; CCL 81) monolayers in T25 flasks and incubated at 35°C for 1 hour to allow for virus attachment. The cultures were then refed with Eagle's minimum essential medium (EMEM) + 2% heat-inactivated fetal bovine serum (FBS; Gibco, Grand Island, NY) and incubated at 35°C for 7 days. After a complete media change on day 7, the cultures were inspected for cytopathic effects (cpes), incubated for an additional 7 days, and reinspected for cpes. Subsequently, the supernatants were harvested, filtered through 0.22-μm filters, mixed 1:1 with heat-inactivated FBS, and stored at −80°C before performing a plaque assay as previously described.^47^ The presence of any virus plaques was scored as a positive viremia result. For the time points for which the samples were found positive, a fresh serum sample was subjected to a standardized cell culture infectivity dose 50 (CCID_50_) assay (De La Barrera R, unpublished data) to quantify the amount of infectious virus. Briefly, the selected serum samples were serially diluted (one initial 10-fold dilution followed by six 3-fold endpoint dilutions) in EMEM-based growth medium with 10% FBS and then inoculated onto wells of Vero (World Health Organization) cell monolayers seeded in 96-well half-area plates. Six days post-inoculation, the cells were fixed with ethanol:methanol (1:1) at −20°C, and a purified, cross-reactive anti-DENV monoclonal antibody conjugated to 6B6-C1-horseradish peroxidase was used to detect DENV-1 and DENV-2 titers using immunocytochemistry. The percentage of positive virus wells per dilution was detected using True Blue chromagenic peroxidase substrate (KPL, Gaithersburg, MD), which selectively stained only the positive wells. Virus titers (expressed as log CCID_50_ per milliliter; calculated using the Spearman—Karber equation) were defined as the reciprocal of the virus dilution resulting in 50% positive wells. The limit of quantitation (LOQ) was defined at 1.0 log_10_ CCID_50_/mL.

### RT-PCR assay.

Viral RNA was extracted from 140 μL each serum sample using the QIAamp Viral RNA Mini Kit (Qiagen, Germany) according to the manufacturer's instructions. DENV replication was determined by quantification of DENV-1 and DENV-2 genomic copies by RT-PCR using a method modified from Sadon and others,^48^ and the AgPath-ID One-Step RT-PCR Kit (Ambion, Austin, TX). Each RT-PCR reaction mixture contained 2.5 μL RNA template (corresponding to approximately 400 ng RNA per sample), 10 pmol either DENV-1 or DENV-2 forward/reverse primers, 5 pmol either DENV-1 or DENV-2 probes, 1.67 μL Detection Enhancer (from the kit), 20 U RNAse inhibitor (RNAsin; Promega, Madison, WI), 1× RT-PCR Buffer, and 1× RT-PCR Enzyme Mix (from the kit) in a total volume of 25 μL. Primers and probes used were: DENV-1, forward 5′-GCA-TTY-CTA-AGA-TTT-CTA-GCC-ATA-CC-3′, reverse 5′-TCG-CTC-CAT-TCT-TCT-TGA-ATG-AG-3′, probe 5′-AAC-AGC-AGG-AAT-TTT-3′ (5′-FAM, 3′-MGB); DENV-2, forward 5′-CTG-CAR-GGA-CGA-GGA-CCA-TT-3′, reverse 5′-GGG-ATT-GTT-AGG-AAA-CGA-AGG-A-3′, probe 5′-AAA-CTG-TTC-ATG-GCC-CTG-GTG-GCR-3′ (5′-Yakima Yellow/3′-Darkquencher). The single-step RT-PCR (consisting of a 10-minute RT step at 45°C and 10 minutes of Taq polymerase activation at 95°C followed by 40 cycles of PCR at 95°C for 15 seconds and at 60°C for 1 minute) was performed on an ABI 7300 Real-Time PCR System (Applied Biosystems, Foster City, CA). A sample was defined as positive if the cycle threshold (Ct) level was < 40 and the LOQ for the RT-PCR assay was determined to be 360 genome equivalents (GEQ) per 1 mL for both DENV-1 and DENV-2.

### Statistics.

All analyses were done using SAS, version 9.2. Statistical analyses were performed to compare NAb (MN_50_) responses from studies 1 and 2 between vaccine groups at different post-vaccination time points using a repeated analysis of variance (ANOVA) model on the log values, with group, time point, and interaction between group and time point as factors. If no significant interaction between group and time point was detected, between-group comparisons were performed overall using the repeated ANOVA model; otherwise, between-group comparisons were performed at each time point. *P* values were adjusted for multiple comparisons using Tukey's method for study 2, and Dunnett's adjustment was used for comparisons with the alum group.

Differences in RNAemia between adjuvanted vaccine groups and the PBS group from study 2 were evaluated using an ANOVA model by challenge for the number of days of RNAemia, a log-rank test for the time to onset of RNAemia, and a non-parametric analysis (ANOVA on ranks) for the mean and peak levels of RNAemia over the 14 days post-challenge. Because the intention was to compare the responses in the adjuvanted vaccine groups with those in the PBS group, Dunnett's adjustment for multiple comparisons was used for all analyses. The level of significance was set at *P* < 0.05.

Pearson correlations between log-transformed, pre-challenge MN_50_ levels for serotypes 1 and 2, duration of RNAemia (greater than LOQ), and log-transformed, maximum RNAemia levels measured over 14 days post-challenge were computed over all groups separately for each challenge.

## Results

### Immunogenicity and reactogenicity of TDENV PIV formulations.

Two studies were performed to evaluate the immunogenicity of two vaccine doses of different TDENV PIV formulations in rhesus macaques by assessment of vaccine-induced NAb responses against all four DENV serotypes. In both studies, all monkeys tolerated the two vaccine doses without obvious systemic or local injection-site reactivity (Supplemental Table 1).

The first study evaluated formulations containing 0.5 or 0.125 μg per serotype antigen adjuvanted with alum or an Adjuvant System (AS01_E_, AS03_A_, or AS04_D_), as well as formulations containing 2.0 μg per serotype, either with alum or without adjuvant.

**Figure 1.**
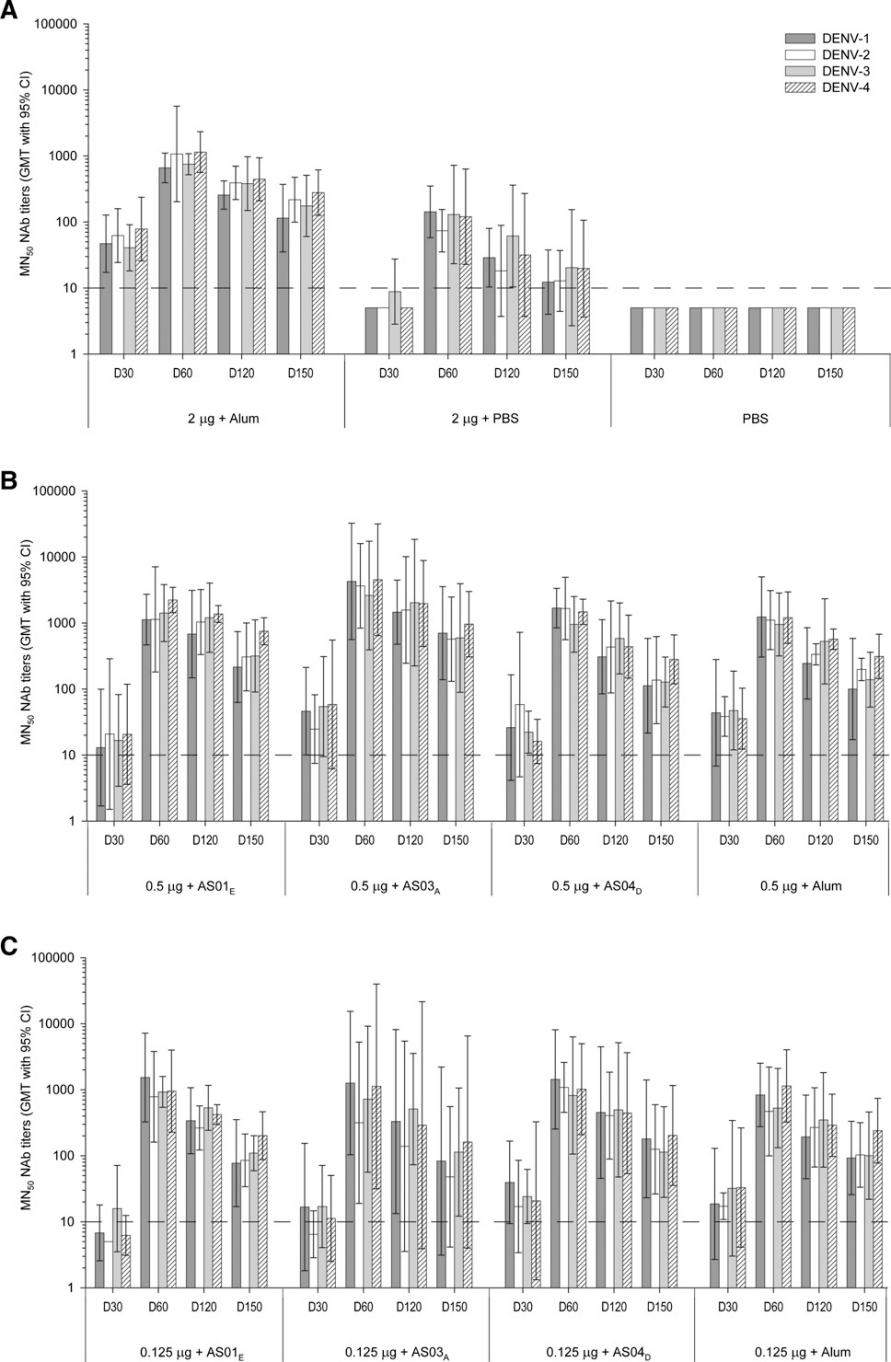
NAb responses to adjuvanted and non-adjuvanted TDENV PIV formulations containing one of three different antigen doses. NAb titers were measured by an ELISA-based MN_50_ assay in serum samples from monkeys who received two doses, 1 month apart, of either an adjuvanted or non-adjuvanted TDENV PIV formulation containing (**A**) 2.0, (**B**) 0.5, or (**C**) 0.125 μg per serotype per dose or PBS (*N* = 4 per group). Sera for the selected time points shown were obtained before (day 30) and 1, 3, or 4 months post-second immunization (days 60, 120, and 150, respectively). The dashed line represents the cutoff value for a positive result. CI = confidence interval; D = day.

One month after the first dose (day 30), all adjuvanted vaccine groups, except the AS01_E_ group with 0.125 μg per serotype, exhibited NAb responses against each of the four DENV serotypes, with group geometric mean titers (GMTs) at or above the assay cutoff (Figure 1). By 1 month after the second dose (day 60), GMTs for all vaccine groups (adjuvanted or non-adjuvanted) had increased at least 10-fold, with titers that were comparable between DENV serotypes. Although there was a decline in GMTs from day 60 onward, all animals remained seropositive until the end of the observation period (day 150).

One month after the second dose, the group that received 0.5 μg per serotype with AS03_A_ exhibited the highest GMTs (> 2,500). When the 0.5- and 0.125-μg doses were compared across days 60, 120, and 150, we observed that, in the AS01_E_ group, DENV-4–specific responses were significantly higher (approximately threefold) for the 0.5-μg dose than for the 0.125-μg dose (*P* = 0.005). Supporting these results, we also detected trends for higher responses with the 0.5-μg dose in the AS01_E_ group for DENV-2 and DENV-3 (geometric mean ratio [GMR] > 2; *P* < 0.10). Comparing 0.5- and 0.125-μg doses in the AS03_A_ group, the GMRs were higher (i.e., > 4), but this was not statistically significant.

When the responses of Adjuvant System-containing vaccines with 0.125- or 0.5-μg antigen doses were each compared with (antigen dose-matched) alum-adjuvanted vaccine across days 60, 120, and 150, there were no significant differences (*P* > 0.05) between the AS04_D_ groups and the alum groups for both antigen doses tested. In the same analysis, it was observed that, for the AS03_A_ groups, the 0.5-μg dose (for DENV-1– and DENV-4–specific responses; *P* = 0.041 and *P* = 0.024, respectively) but not the 0.125-μg dose was associated with higher NAb responses compared with the alum groups.

In conclusion, because there was no difference between the AS04_D_ and alum groups after two immunizations, additional testing of AS04 (which contains alum and MPL) was not justified, and alum was retained in the subsequent study as the benchmark adjuvant. There was an overall trend (supported by significant and non-significant results) for higher NAb responses with the 0.5-μg per serotype antigen dose compared with the 0.125-μg per serotype antigen dose. Collectively, these results supported additional preclinical evaluation of formulations containing the 0.5-μg per serotype antigen dose with alum, AS01, or AS03.

In a second study, we evaluated NAb responses elicited by TDENV PIV formulations containing 0.5 μg per serotype adjuvanted with alum, AS01_E_, or one of three different dose levels of AS03 (AS03_A_, AS03_B_ or AS03_C_; in decreasing order). Titers for individual animals are presented in Supplemental Table 2.

**Figure 2.**
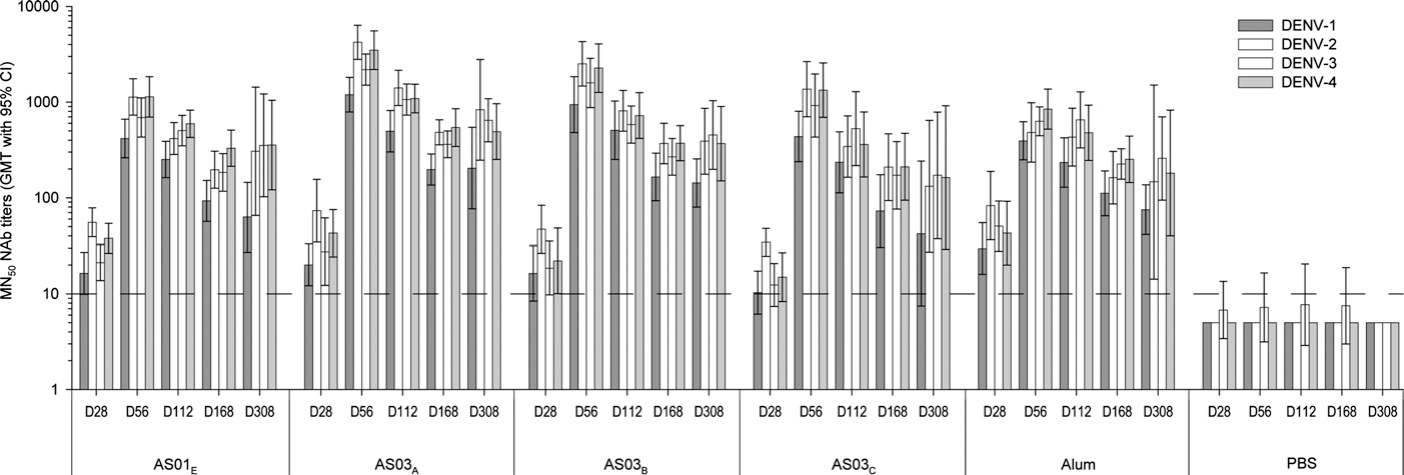
NAb responses to adjuvanted TDENV PIV formulations containing 0.5 μg antigen per serotype. NAb titers were measured by an ELISA-based MN_50_ assay in serum samples from monkeys who received two doses, 1 month apart, of either an adjuvanted TDENV PIV formulation (0.5 μg per serotype per dose), or PBS. Sera for the selected time points shown were obtained before (day 28) and 4, 12, 20, and 40 weeks after the second immunization (days 56, 168, and 308, respectively; *N* = 10 per group on days 28, 56, and 168 and *N* = 5 per group on day 308). The dashed line represents the cutoff value for a positive result. CI = confidence interval; D = day.

All vaccine groups exhibited seroconversion against each of the four DENV serotypes after the first dose (day 28), and GMTs peaked 1 month after the second dose on day 56. During the entire post-vaccination follow-up period, all groups were significantly different from the PBS group for all DENV serotypes (Figure 2). Although GMTs for each DENV serotype in all groups declined from day 56 to day 168, responses were persistent and remained remarkably stable between days 168 and 308. GMTs were comparable between the latter two time points (*P* > 0.05), except for the GMTs for DENV-3 in the AS03_A_, AS03_B_, and AS01_E_ groups, which were higher on day 308 relative to day 168 (*P* < 0.05). Across time points and groups, GMTs against DENV-1 were significantly lower (*P* < 0.01) than those against DENV-2, -3, and -4 (GMR for DENV-1 compared with DENV-2, -3, and -4: 0.3–0.6). The GMTs for DENV-2, -3, and -4 were comparable at all time points, with the exception of relatively minor differences on day 28 (GMT for DENV-3 < GMTs for DENV-2 and -4; GMR = 0.7), day 168 (GMT for DENV-4 > GMT for DENV-2 and -3; GMR = 1.3), and day 252 (GMT for DENV-4 > GMT for DENV-2; GMR 1.4; *P* < 0.05).

Similar to the results of the first study, the AS03_A_ group exhibited the highest GMTs. Indeed, for this group, GMTs against DENV-2 were significantly higher (at least threefold) on days 56 and 112 compared with those of the groups receiving formulations with alum (*P* ≤ 0.014), AS03_C_ (*P* ≤ 0.021), or AS01_E_ (*P* ≤ 0.010), and on day 56, the GMTs against DENV-3 for the AS03_A_ group were significantly higher (approximately threefold) than for the alum and AS01_E_ groups (*P* = 0.004 and *P* = 0.010, respectively). Moreover, across time points, vaccine-induced NAb responses against DENV-1 were significantly (approximately threefold) higher for the AS03_A_ group than for the AS03_C_ group (*P* = 0.034), suggesting a dose response for AS03. However, with respect to DENV-4–specific responses, no significant differences were detected between any of the three AS03 groups or between these groups and the alum and AS01_E_ groups.

### Challenge with live DENV-1 and DENV-2.

Animals from the second study were challenged with either low-passage DENV-2 S16803 at 32 weeks post-dose 2 (day 252) or low-passage DENV-1 PR/94 at 40 weeks post-dose 2 (day 308). These DENV strains were selected on the basis of a history of reliable and robust viremia (the presence of infectious virus) in naïve rhesus macaques. Sera obtained daily for 14 days post-challenge were tested for viremia and viral RNA (RNAemia). Data from individual animals are presented in Supplemental Table 2.

**Figure 3.**
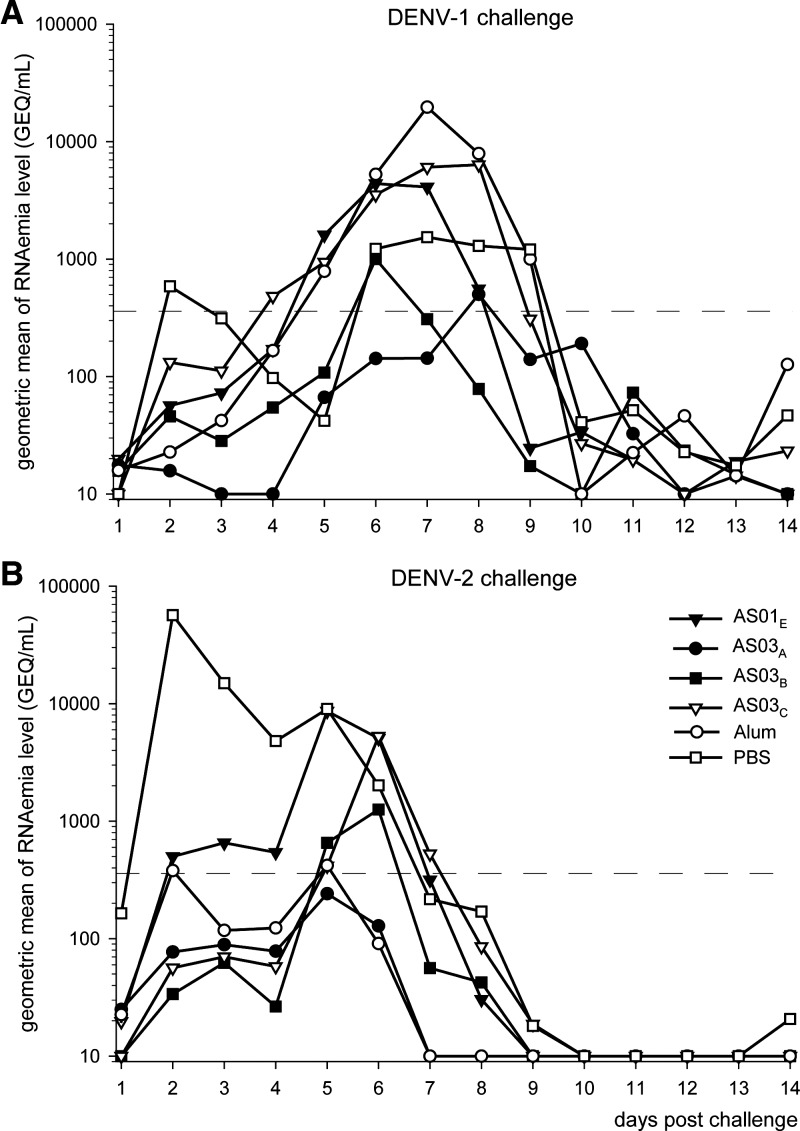
Circulating DENV genome (RNAemia) in serum post-challenge with either DENV-1 or DENV-2. Serum samples from monkeys (*N* = 5 per group) who received an adjuvanted TDENV PIV formulation (0.5 μg per type per dose) or PBS were obtained daily post-challenge with either (**A**) DENV-1 or (**B**) DENV-2 and tested for the presence of viral RNA (RNAemia) by RT-PCR assay. The dashed line represents the limit of quantitation (i.e., 360 GEQ/mL; therefore, values below this limit are approximations).

In the negative control (PBS) groups, the DENV-1 and DENV-2 challenge strains induced 7.4 and 5.2 mean days of viremia with 9 and 7 days of detectable RNAemia, respectively (Figure 3 and Table 1). This showed that the challenge was successful for both DENV serotypes. After DENV-2 challenge, there was no measurable viremia in any of the vaccine groups, and after DENV-1 challenge, there were only 0.2 mean days of viremia (corresponding to 1 day of viremia in one of five animals) without quantifiable amounts of serum virus in the groups that received TDENV PIV formulated with AS03_A_ or alum. Although both challenge viruses induced RNAemia in each of the vaccine groups, duration was reduced compared with that in the control group, and this reduction was significant or nearly significant for the AS03_A_ and AS03_B_ groups after DENV-1 challenge (*P* = 0.024 and *P* = 0.053, respectively; both approximately 3 days decrease). Similarly, compared with controls, the duration of RNAemia in the alum, AS03_A_, and AS03_B_ groups was significantly reduced after DENV-2 challenge (*P* = 0.024, *P* = 0.017, and *P* = 0.024, respectively; all approximately 4 days decrease). In addition, the onset of RNAemia after DENV-2 challenge seemed to be slightly delayed in the AS03_C_ group compared with the control group (*P* = 0.011). Otherwise, there were no significant differences with the controls in the time to onset of RNAemia or the mean serum RNA titers over the 14 days post-challenge for either virus. In contrast, the maximum RNAemia levels in the alum group after DENV-1 challenge were significantly higher than in the control group (*P* = 0.043; group medians were 14,300 and 2,720 GEQ/mL, respectively). The maximum RNAemia levels in the AS01_E_ and AS03_C_ groups (medians of 9,210 and 5,730 GEQ/mL, respectively) were not significantly different from the PBS control group. Unlike the maximum RNAemia levels after DENV-1 challenge, the levels after DENV-2 challenge in each of the vaccine groups were not significantly different from those in the control group (*P* > 0.05).

**Figure 4.**
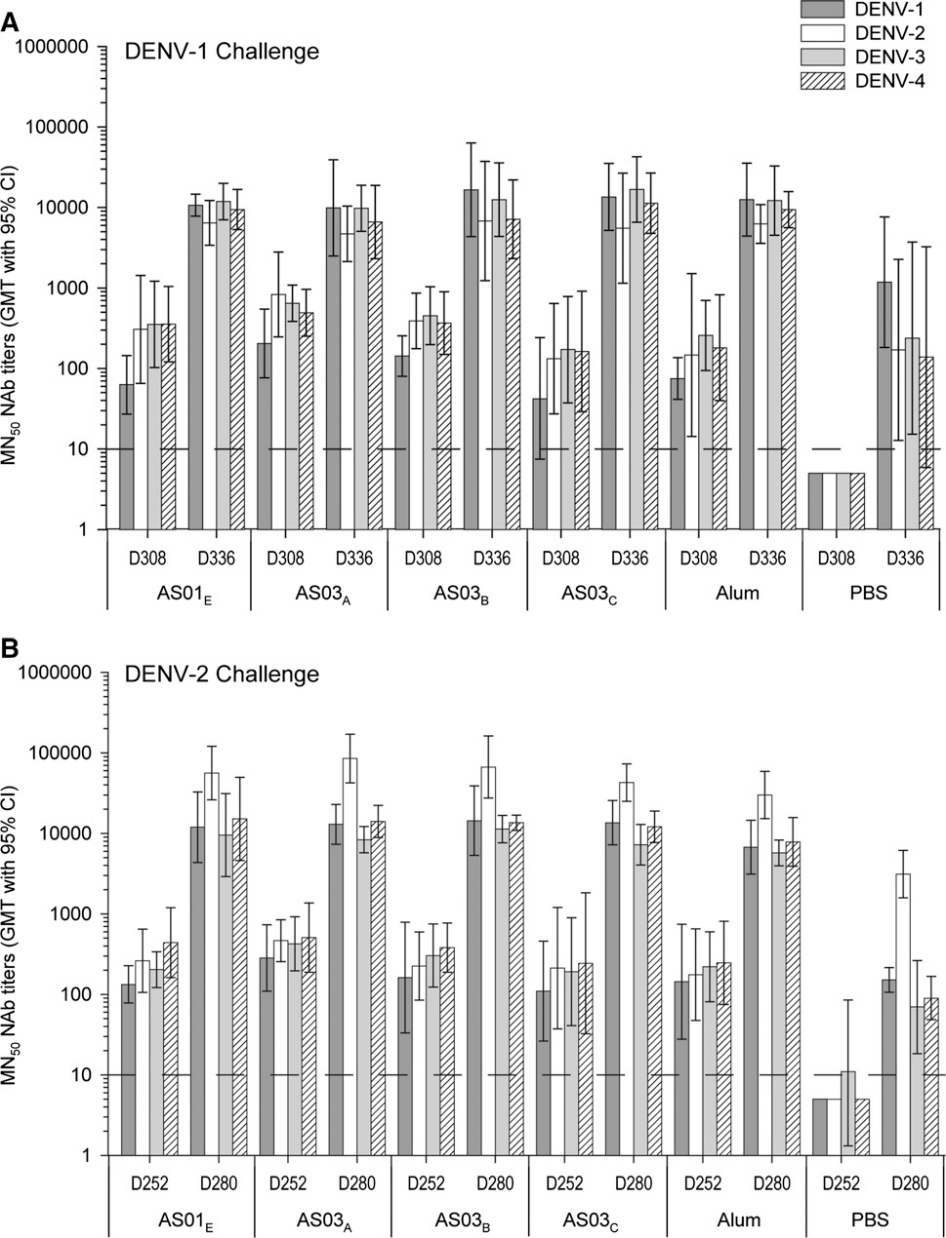
NAb responses before and after viral challenge with DENV-1 or DENV-2. NAb titers were measured by an ELISA-based MN_50_ assay in serum samples from monkeys (*N* = 5 per group) who received either an adjuvanted TDENV PIV formulation (0.5 μg per type per dose) or PBS, and were challenged at 40 (day 308) or 32 (day 252) weeks post-second vaccination with either (**A**) DENV-1 or (**B**) DENV-2, respectively. Sera were obtained at the days of challenge and 1 month later. The dashed line represents the cutoff value for a positive result. CI, confidence interval; D = day

Sera from vaccinated animals obtained 1 month after challenge showed a robust and long-lasting anamnestic antibody response to the DENV-1 and DENV-2 challenge viruses (Figure 4). The titers of the antibody responses against each of the four serotypes were at least 10-fold higher than in the unvaccinated controls. This suggests that these animals were primed to respond to epitopes present on the challenge virus strains. There were no significant differences between the vaccine groups when comparing the increase in GMTs from the first to the second month post-challenge for either challenge DENV serotype.

There was no significant correlation (*P* > 0.05) between the DENV-1– and DENV-2–specific NAb titers at the day of challenge and the maximum RNAemia levels after the challenge with DENV-1 (Supplemental Figure 1A). However, there was a weak but statistically significant negative correlation between DENV-1 and DENV-2 NAb titers on the day of challenge and maximum RNAemia levels after challenge with DENV-2 (*P* ≤ 0.037; Pearson's *r* was approximately −0.4). There was also a moderate, statistically significant negative correlation (Pearson's *r* was approximately −0.5; *P* ≤ 0.005) between the DENV-1 and DENV-2 NAb titers at the day of challenge and the number of days of quantifiable (greater than LOQ) RNAemia, irrespective of the challenge virus used (Supplemental Figure 1B).

## Discussion

Dengue has a significant public health impact worldwide, and there is an unmet need for a safe and effective vaccine. This study was performed to evaluate a novel TDENV PIV vaccine candidate for immunogenicity and ability to restrict viral replication after DENV challenge in the rhesus macaque infection model. The results show that vaccine formulations with alum or an Adjuvant System were well-tolerated and induced robust and persistent NAb responses to all four DENV serotypes 1 month after the second immunization.

Although NAb responses against all four DENV serotypes were detected, the GMTs against DENV-1 were approximately twofold lower than those against the other DENV serotypes. However, the biological relevance of this is not known because of the lack of an established NAb correlate/surrogate of protection. Although GMTs declined from their peak at 1 month post-dose 2 onward, the rate of decline seemed to decrease over time, and responses remained detectable and stable from day 168 to day 252 or day 308 (i.e., the latest time points before challenge with DENV-2 or DENV-1, respectively). The responses against each of the four serotypes were strongly boosted by the challenge viruses, indicating that immunity to challenge virus was not sterile on days 258 or 308.

Clinical observation of the NHPs after immunization suggests that the vaccine antigens, either alone or combined with one of the adjuvants tested, were not associated with adverse effects. Because TDENV PIV does not replicate, we hypothesized that an adjuvant might be necessary to enhance its immunogenicity, particularly at lower antigen doses. Indeed, each of the adjuvants tested seemed to enhance the immune response to the viral antigens. Compared with the non-adjuvanted vaccine, the adjuvanted vaccines elicited robust anamnestic NAb responses against each DENV serotype after dose 2, with peak GMTs that were up to 10-fold higher and titers that persisted at higher levels after vaccination, thus showing an adjuvant effect. In two independent studies, a tendency toward higher titers was observed in animals that received an antigen dose of 0.5 μg per serotype formulated with AS03_A_. AS03 has also been shown to enhance the immunogenicity of influenza vaccines.^49^–^52^ For the current DENV antigens (at a 0.5-μg per serotype dose), AS03 and AS01 seemed to be more potent than alum or AS04 at inducing robust NAb responses in rhesus macaques after two doses.

After challenge with DENV-1 or DENV-2 strains, all vaccinated animals but not those of the control groups exhibited complete or near-complete protection against DENV-1 and DENV-2 viremia (i.e., infectious virus in serum detected in Vero cells). However, most animals in the control and vaccine groups had detectable viral RNA in their serum (RNAemia), and one vaccinated group (the alum group) had a significantly higher DENV-1 RNAemia peak than the control group. The mechanism responsible for the enhanced DENV-1 RNAemia in the alum-vaccinated group is not known. However, there seemed to be a discordance between these results and our inability to isolate infectious virus in Vero cell culture. There are several possible explanations for these results. The positive RT-PCR signals could be caused by detection of viral RNA in the inoculum without challenge virus replication (i.e., RNA derived from degraded viruses), which we consider less likely, because RT-PCR titers increased over several days. Alternatively, these signals could be caused by limited challenge virus replication. Low-level viremia might not be detectable in Vero cell culture owing to the presence of NAbs. The rapid anamnestic responses to the challenge viruses and resulting high titered virus NAbs in the sera might have prevented our ability to detect low levels of viremia in Vero cells. The increase in NAb responses is consistent with this interpretation of the viral RNA data. To explore this further, we are attempting to isolate infectious virus from immune complexes by inoculation of viral RNA-positive sera obtained from this study onto Fcγ-receptor (FcγR) -bearing cells in culture. The possibility that at least some of the viral RNA measured after challenge is present in neutralized virus–antibody complexes has also been proposed to explain similar results in a previous study with a monovalent DENV-2 PIV.^36^ In the same study, a similar discordance between RNA and viremia was also reported with another vaccine candidate, an adjuvanted recombinant subunit E protein vaccine.^36^ In addition, in a recent efficacy study in cynomolgus macaques, several animals vaccinated with a live-attenuated tetravalent chimeric DENV vaccine candidate (DENVax) exhibited serum RNA titers of up to 6.3 log_10_ GEQ/mL after virus challenge, but little or no detectable live virus when measured on Fc receptor-negative cells.^24^ Collectively, these data suggest that differences between RNAemia and viremia results may be a common observation for both live as well as non-replicating DENV vaccine candidates.

Sterilizing immunity, characterized by the absence of an increase in NAb titers as well as absence of detectable viremia after challenge, has been reported by several groups after vaccination of rhesus macaques with DENV LAV.^36^,^53^ Although the ability of a DENV vaccine to induce sterilizing immunity may be desirable, the duration of this state may be relatively short-lived. In a previous study, rhesus macaques vaccinated with a DENV-2 LAV initially showed sterilizing immunity to DENV-2 challenge but exhibited strong anamnestic antibody responses to DENV-2 on being rechallenged with the same virus 1 year later (Putnak J. R. and others, unpublished data). The maintenance of generally high DENV NAb titers observed in individuals living in dengue-endemic areas also suggests the possibility for asymptomatic or inapparent reinfections resulting in anamnestic-type antibody responses.^11^,^54^ Therefore, the ability to induce sterilizing immunity and/or to induce memory B cells that can be activated quickly upon re-exposure to infecting viruses and differentiate into long-lived bone marrow plasma cells, each represents a desirable feature of a DENV vaccine. In this study, all vaccinated animals mounted robust anamnestic antibody responses to epitopes on the challenge viruses, and this may play a critical role in abrogating virus replication and preventing disease. In addition, a role for vaccine-induced CMI in the protective immune response against subsequent DENV challenge cannot be ruled out. Although NAb responses are critical for blocking infection, T cell-mediated immunity could play a role in reducing the viral load after infection (reviewed in refs. 27 and 55). However, neither antibody nor CMI correlates of protective immunity against DENV has yet been established, and both are subject to ongoing debate and study.

Although DENV NAbs are widely considered to be a potential primary correlate of protection against dengue disease,^7^ no protective threshold titer similar to the minimum protective titer of 1:10 for Japanese encephalitis virus has yet been established for DENV.^56^,^57^ In this study, the absence of viremia seemed to coincide with the presence of NAb titers (> 10) at the time of challenge. A negative correlation with NAb titers was also observed for maximum RNAemia levels post-challenge with DENV-2 (but not with DENV-1) and with the number of days of quantifiable (> LOQ) RNAemia, irrespective of the challenge virus used. In addition, several adjuvanted vaccine groups exhibited both high-titered NAb responses at the time of challenge as well as significantly fewer days of RNAemia relative to the control group. Additionally, it is possible that high antibody avidity is an important feature for protection, as suggested in a previous study in rhesus macaques with an alum-adjuvanted TDENV PIV.^38^ In this study, Simmons and others^38^ reported that the total antiviral antibody titers (as measured by ELISA) along with high antibody avidity at the time of challenge correlated more strongly with reduction in viremia than NAb titers. The requirement for antibodies of a certain quality is also suggested by results from the recent phase 2b efficacy trial of the CYD(1–4) live-attenuated dengue vaccine, which failed to showed protection against DENV-2, although it elicited high-titered DENV-2 NAb responses.^20^ One of the several possible explanations for this is that, at a given NAb titer, cross-reactive antibodies may be less protective than serotype-specific antibodies, as suggested in previous epidemiological studies.^54^ Moreover, NAb titers have been used as a marker for a protective immune response against flaviviruses, irrespective of whether the actual mechanisms of protection are based on cellular responses, humoral responses, or a combination of both.^58^

## Conclusions

When tested in an NHP infection model, adjuvanted TDENV PIV vaccine formulations showed an acceptable safety profile and were highly immunogenic, with all formulations inducing robust and persistent NAb responses against each of the four DENV serotypes. Although the increase in RNAemia observed over time and the presence of anamnestic NAb responses after challenge suggest replication of the challenge viruses, the nearly complete absence of detectable infectious virus suggests that a protective immune response was induced. TDENV PIV vaccine candidates formulated with alum or an Adjuvant System can, therefore, be considered appropriate for clinical evaluation, and phase 1 studies are currently underway (NCT01666652 and NCT01702857).

## Supplementary Material

Supplemental Datas.

## Figures and Tables

**Table 1:** Post-challenge viremia, RNAemia, and NAb responses in monkeys immunized with adjuvanted TDENV PIV formulations

Formulation*	Viremia				RNAemia				NAb response specific for challenge virus (GMT)			
	DENV-1 challenge		DENV-2 challenge		DENV-1 challenge		DENV-2 challenge		DENV-1 challenge		DENV-2 challenge	
	Duration†	Max titer‡	Duration†	Max titer‡	Duration†	Max titer§	Duration†	Max titer§	Day 308¶	Day 336	Day 252¶	Day 280
Control (PBS)	7.4	1.7	5.2	3.2	9.0	2,720	7.0	85,800	< 10	1,182	< 10	3,125
TDENV PIV/AS01_E_	0	nd	0	nd	7.0	9,210	5.4	7,360	63	10,676	262	56,074
TDENV PIV/AS03_A_	0.2	< 1.0	0	nd	4.6	1,830	2.4	3,320	204	9,890	465	85,228
TDENV PIV/AS03_B_	0	nd	0	nd	5.4	1,260	2.8	12,500	143	16,593	225	66,795
TDENV PIV/AS03_C_	0	nd	0	nd	8.0	5,730	3.8	9,830	42	13,526	212	42,885
TDENV PIV/Alum	0.2	< 1.0	0	nd	7.8	14,300	2.6	1,320	75	12,534	176	30,018

nd = non-detectable viremia.

* All TDENV PIV formulations contained 0.5 μg antigen per DENV serotype.

† Viremia and RNAemia durations are expressed as the group mean number of days with viremia, and with detectable RNAemia (Ct < 40), respectively.

‡ Maximum viremia titers represent the highest individual titers measured over the 14 days post-challenge per group, in log_10_ of the CCID_50_ per 1 mL; < 1.0 are results below the limit of quantitation of 1.0 log_10_ CCID_50_/mL.

§ Maximum RNAemia titers represent the group medians of the maximum titers over the 14 days post-challenge per animal, expressed in GEQ per 1 mL.

¶ Days 308 and 252 were the days of viral challenge with DENV-1 or DENV-2, respectively.
