# Supplementary material for: An Adjuvanted, Tetravalent Dengue Virus Purified Inactivated Vaccine Candidate Induces Long-Lasting and Protective Antibody Responses Against Dengue Challenge in Rhesus Macaques
Source: Am J Trop Med Hyg. 2015 Apr 1;92(4):698–708. doi: 10.4269/ajtmh.14-0268 (PMC4385761; doi:10.4269/ajtmh.14-0268)
Supplement: Supplementary file 1 [file SD1.pdf]

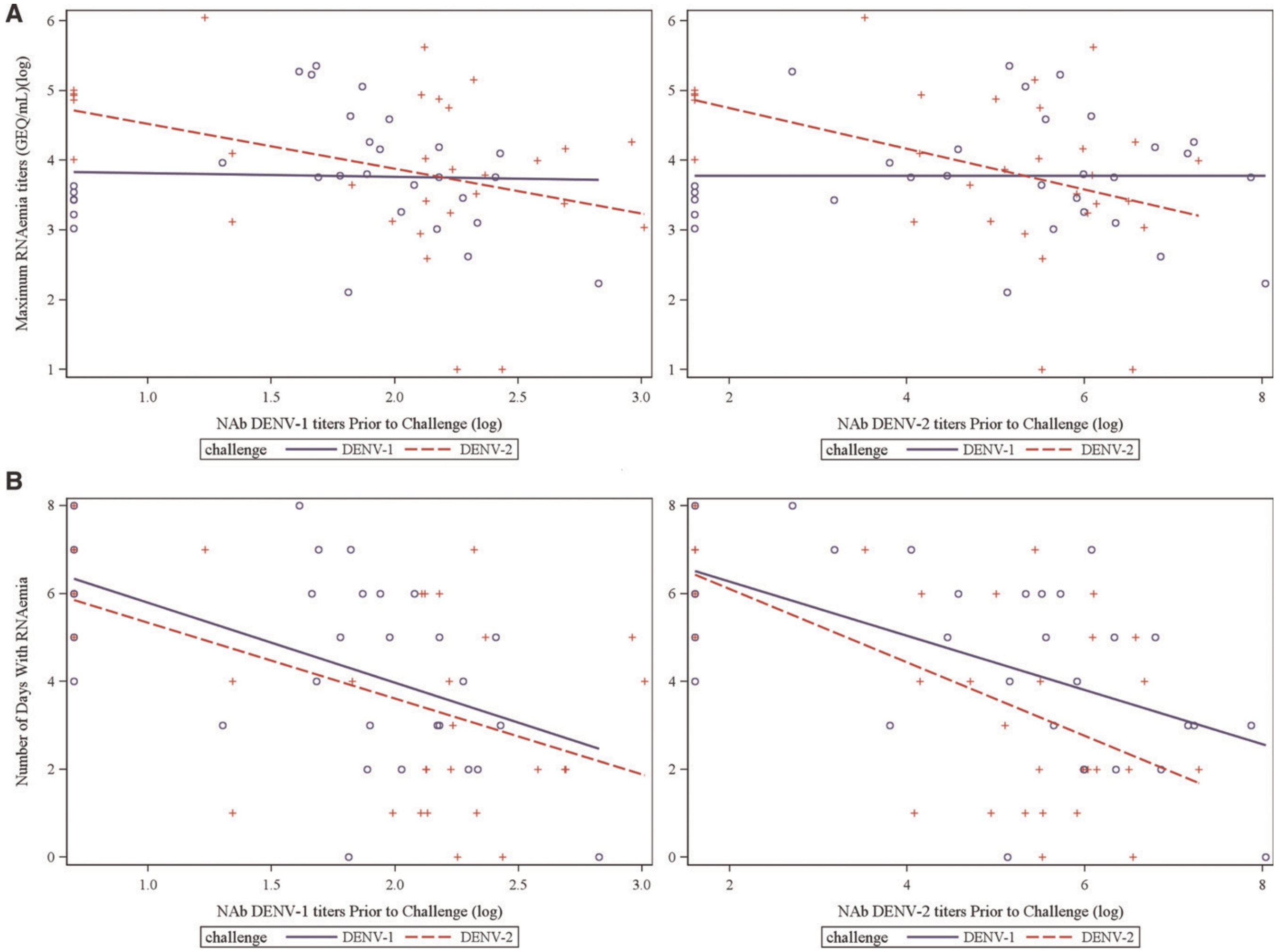

Supplemental Figure 1. Regression plots showing the correlation between the log neutralizing antibody (NAb) titers at the day of challenge with dengue virus-1 (DENV-1) or DENV-2 and (**A**) the maximum RNAemia levels (in genome equivalents [GEQ] per 1 mL) post-challenge with DENV-1 or DENV-2 or (**B**) the duration of RNAemia (number of days with RNAemia exceeding the limit of quantitation).

Supplemental Table 1

Vaccine reactogenicity: local reactogenicity during days 0–3 post-vaccination and systemic reactogenicity during days 0–7 post-vaccination

	Study 1											Study 2					
Group	2 μg + Alum	2 μg + PBS	0.5 μg + $AS01_E$	0.5 μg + $AS03_A$	0.5 μg + $AS04_D$	0.5 μg + Alum	0.125 μg + $AS01_E$	0.125 μg + $AS03_A$	0.125 μg + $AS04_D$	0.125 μg + Alum	PBS	$AS01_E$	$AS03_A$	$AS03_B$	$AS03_C$	Alum	PBS
N	4	4	4	4	4	4	4	4	4	4	4	10	10	10	10	10	10
Age range (years)	5–9	5–10	5–9	6–11	5–10	5–14	5–9	5–10	5–9	6–11	6–10	5–10	5–9	4–9	5–8	5–9	5–9
Sex ratio (male/female)	3/1	3/1	3/1	2/2	3/1	3/1	3/1	3/1	2/2	2/2	3/1	4/6	1/9	5/5	2/8	6/4	7/3
Weight (kg)	7–9	7–10	6–10	5–8	8–11	8–11	6–13	5–10	6–8	7–10	7–11	6–12	5–11	5–12	5–13	5–16	6–14
Local																	
Post-dose 1																	
Redness and bruising																	
All	1	1	1	4	5	5	5	1	1	4	3	6	1	0	0	0	0
Grade 1	1	1	1	4	5	5	5	1	1	1	3	4	0	0	0	0	0
Grade 2	0	0	0	0	0	0	0	0	0	0	0	2	1	0	0	0	0
Grade 3	0	0	0	0	0	0	0	0	0	3	0	0	0	0	0	0	0
Induration																	
All	0	0	2	1	2	0	1	1	0	0	2	6	0	3	8	4	1
Grade 1	0	0	1	0	0	0	0	0	0	0	0	0	0	0	0	0	0
Grade 2	0	0	1	1	2	0	1	1	0	0	2	1	0	2	8	4	1
Grade 3	0	0	0	0	0	0	0	0	0	0	0	5	0	1	0	0	0
Swelling																	
All	0	0	0	1	0	0	1	0	0	0	0	1	0	0	1	0	0
Grade 1	0	0	0	0	0	0	0	0	0	0	0	0	0	0	0	0	0
Grade 2	0	0	0	0	0	0	0	0	0	0	0	1	0	0	1	0	0
Grade 3	0	0	0	1	0	0	1	0	0	0	0	0	0	0	0	0	0
Post-dose 2																	
Redness & bruising																	
All	4	0	1	3	0	1	0	0	3	0	0	4	1	3	2	0	3
Grade 1	1	0	1	3	0	1	0	0	3	0	0	4	1	3	2	0	1
Grade 2	0	0	0	0	0	0	0	0	0	0	0	0	0	0	0	0	0
Grade 3	3	0	0	0	0	0	0	0	0	0	0	0	0	0	0	0	2
Induration																	
All	0	0	0	0	0	3	0	0	0	0	0	4	10	3	8	3	3
Grade 1	0	0	0	0	0	0	0	0	0	0	0	0	0	0	0	0	0
Grade 2	0	0	0	0	0	3	0	0	0	0	0	3	1	1	5	2	3
Grade 3	0	0	0	0	0	0	0	0	0	0	0	1	9	2	3	1	0
Swelling																	
All	0	0	0	0	0	0	0	0	0	0	0	0	1	0	0	0	0
Grade 1	0	0	0	0	0	0	0	0	0	0	0	0	1	0	0	0	0
Grade 2	0	0	0	0	0	0	0	0	0	0	0	0	0	0	0	0	0
Grade 3	0	0	0	0	0	0	0	0	0	0	0	0	0	0	0	0	0
Systemic																	
Pre-vaccination																	
All	1	1	0	3	1	5	0	1	9	5	0	25	31	22	36	39	28
Grade 1	1	1	0	3	1	5	0	1	9	5	0	23	26	15	22	32	27
Grade 2	0	0	0	0	0	0	0	0	0	0	0	2	5	6	11	6	1
Grade 3	0	0	0	0	0	0	0	0	0	0	0	0	0	1	3	1	0
Post-dose 1																	
All	11	1	0	7	2	6	1	1	5	10	2	35	50	39	52	37	38
Grade 1	8	1	0	6	2	6	1	1	5	7	2	18	26	22	27	20	15
Grade 2	2	0	0	1	0	0	0	0	0	1	0	9	15	7	16	9	13
Grade 3	1	0	0	0	0	0	0	0	0	2	0	8	9	10	9	8	10

(continued)

Supplemental Table 1
Continued

Group	Study 1											Study 2					
	2 μg + Alum	2 μg + PBS	0.5 μg + $AS01_E$	0.5 μg + $AS03_A$	0.5 μg + $AS04_D$	0.5 μg + Alum	0.125 μg + $AS01_E$	0.125 μg + $AS03_A$	0.125 μg + $AS04_D$	0.125 μg + Alum	PBS	$AS01_E$	$AS03_A$	$AS03_B$	$AS03_C$	Alum	PBS
Post-dose 2																	
All	11	6	1	7	1	3	2	4	8	6	3	33	33	35	40	34	49
Grade 1	6	4	0	5	1	3	2	4	8	6	3	11	18	13	21	14	21
Grade 2	1	2	0	2	0	0	0	0	0	0	0	16	13	13	10	10	16
Grade 3	4	0	1	0	0	0	0	0	0	0	0	6	2	9	9	10	12

Incidence is expressed as the total number of events per group measured daily during days 0–3 post-vaccination for local reactogenicity. 0 = Absence/no evidence of skin redness, bruising, swelling, induration, or any lesion. Redness: grade 1 = mild (pink skin); grade 2 = moderate (red skin); grade 3 = severe (dark red skin). Bruising: grade 1 = bruise < 5 mm; grade 2 = bruise 5–10 mm; grade 3 = bruise > 10 mm. Muscle induration: grade 1 = mild, hardened area with diameter < 5 mm; grade 2 = moderate, hardened area with diameter 5–10 mm; grade 3 = severe, whole area is hardened and obviously enlarged with diameter > 10 mm. Swelling: grade 1 = slightly swollen/larger compared with the unvaccinated leg, affected area < 5 mm; grade 2 = markedly swollen/larger compared with the unvaccinated leg, affected area 5–10 mm; grade 3 = obviously swollen/larger compared with the unvaccinated leg, affected area > 10 mm. Incidence is expressed as the total number of events per group measured daily during days (–7) to (–1) before vaccination and during days 0–7 post-vaccination for systemic reactogenicity. Grade 1 = mildly reduced appetite; grade 2 = moderately reduced appetite; grade 3 = severely reduced appetite/anorexia.

Supplemental Table 2

Post-challenge viremia, RNAemia and Nab responses for individual monkeys immunized with adjuvanted TDENV PIV formulations

Group/monkey ID	Challenge virus	Viremia max titer* (log $CCID_{50}$/mL)	RNAemia max titer† (GEQ/mL)	NAb titers					
				Pre-challenge‡		Post-challenge‡		Post-/pre-challenge§	
				DENV-1	DENV-2	DENV-1	DENV-2	DENV-1	DENV-2
TDENV PIV + AS01$_E$									
R105	DENV-1	nd	9,210	20	45	10,606	8,504	530	189
R252	DENV-1	nd	42,900	66	434	7,654	4,551	116	11
R431	DENV-1	nd	6,340	77	399	10,178	9,766	132	25
R450	DENV-1	nd	4,460	120	249	10,778	3,085	90	12
R525	DENV-1	nd	18,400	79	1,379	15,576	9,441	197	7
TDENV PIV + AS03$_A$									
R143	DENV-1	nd	1,830	106	402	22,784	6,657	215	17
R259	DENV-1	nd	39,000	95	260	8,160	5,827	86	22
R330	DENV-1	< 1.0	420	198	954	3,754	3,388	19	4
R336	DENV-1	nd	171	669	3,094	3,282	1,850	5	1
R546	DENV-1	nd	12,600	267	1,289	41,312	9,466	155	7
TDENV PIV + AS03$_B$									
R111	DENV-1	nd	1,260	216	572	35,535	7,711	165	14
R323	DENV-1	nd	129	65	169	3,536	1,047	54	6
R447	DENV-1	nd	1,040	148	284	9,651	5,024	65	18
R453	DENV-1	nd	2,910	189	369	53,075	49,321	281	134
R608	DENV-1	nd	15,300	151	892	19,542	7,218	129	8
TDENV PIV + AS03$_C$									
R234	DENV-1	nd	226,000	48	173	18,294	901	381	5
R249	DENV-1	nd	5,730	49	57	4,586	4,094	94	72
R255	DENV-1	nd	170,000	46	306	11,494	9,160	250	30
R528	DENV-1	nd	2,680	5	24	12,307	5,252	2,461	219
R601	DENV-1	nd	5,710	256	562	38,151	29,337	149	52
TDENV PIV + Alum									
R207	DENV-1	nd	5,980	60	86	29,137	4,517	486	53
R253	DENV-1	nd	5,720	151	2,626	20,406	11,677	135	4
R413	DENV-1	nd	14,300	87	97	17,377	8,554	200	88
R415	DENV-1	nd	115,000	74	207	8,536	4,975	115	24
R538	DENV-1	< 1.0	187,000	41	15	3,508	4,228	86	282
PBS									
R451	DENV-1	1.7	4,250	5	5	570	29	114	6
R452	DENV-1	1.4	2,720	5	5	563	28	113	6
R501	DENV-1	1.4	1,050	5	5	449	240	90	48
R514	DENV-1	< 1.0	1,670	5	5	967	159	193	32
R522	DENV-1	1.6	3,500	5	5	16,530	4,618	3,306	924
TDENV PIV + AS01$_E$									
R303	DENV-2	nd	419,000	132	446	17,000	54,392	129	122
R316	DENV-2	nd	4,390	67	111	9,417	33,722	141	304
R502	DENV-2	nd	143,000	209	230	39,693	146,744	190	638
R512	DENV-2	nd	2,610	134	664	4,750	31,961	35	48
R602	DENV-2	nd	7,360	171	164	8,011	64,444	47	393
TDENV PIV + AS03$_A$									
R310	DENV-2	nd	3,320	214	369	16,249	41,962	76	114
R324	DENV-2	nd	6,170	232	440	12,056	71,922	52	164
R438	DENV-2	nd	0	273	696	23,836	121,287	87	174
R520	DENV-2	nd	1,080	1,025	791	6,926	179,960	7	228
R604	DENV-2	nd	10,500	133	242	11,310	6,8267	85	282
TDENV PIV + AS03$_B$									
R322	DENV-2	nd	12,500	22	63	5,695	31,457	259	499
R401	DENV-2	nd	880	127	206	16,270	62,194	128	302
R421	DENV-2	nd	14,600	491	397	13,615	218,474	28	550
R513	DENV-2	nd	2,390	488	461	49,750	58,048	102	126
R517	DENV-2	nd	56,200	166	244	9,867	53,588	59	220
TDENV PIV + AS03$_C$									
R251	DENV-2	nd	1,340	98	140	30,612	57,741	312	412
R311	DENV-2	nd	1.11E+06	17	34	12,163	32,007	716	941
R314	DENV-2	nd	1,770	168	417	14,679	24,410	87	59
R509	DENV-2	nd	9,830	378	1,461	7,740	45,658	21	31
R539	DENV-2	nd	75,400	151	149	10,910	70,419	72	473
TDENV PIV + Alum									
R250	DENV-2	nd	18,400	912	716	7,523	26,517	8	37
R301	DENV-2	nd	390	135	251	17,194	62,772	127	250
R312	DENV-2	nd	86,400	128	64	6,795	17,281	53	270
R510	DENV-2	nd	1,320	22	59	4,922	43,514	224	738
R527	DENV-2	nd	0	179	250	3,206	19,472	18	78

(continued)

Supplemental Table 2
Continued

Group/monkey ID	Challenge virus	Viremia max titer* (log $CCID_{50}$/mL)	RNAemia max titer† (GEQ/mL)	NAb titers					
				Pre-challenge‡		Post-challenge‡		Post-/pre-challenge§	
				DENV-1	DENV-2	DENV-1	DENV-2	DENV-1	DENV-2
PBS									
R114	DENV-2	3.2	89,600	5	5	152	2,413	30	483
R305	DENV-2	2.8	101,000	5	5	244	7,844	49	1,569
R506	DENV-2	1.5	10,300	5	5	145	2,900	29	580
R526	DENV-2	2.7	72,600	5	5	117	1,850	23	370
R603	DENV-2	2.7	85,800	5	5	128	2,937	26	587

GEQ = genome equivalent; nd = non-detectable viremia.
*Maximum titer denotes highest viremia titer over the 14 days post-challenge (days 309–322 and days 253–266 for DENV-1 and DENV-2 challenge, respectively) expressed in $\log_{10}$ of the 50% cell culture infective dose ($CCID_{50}$) per 1 mL. < 1.0 = result below the limit of quantitation of 1.0 log10 CCID50/mL.
†Maximum RNAemia titers measured over the 14 days post-challenge.
‡Pre-challenge samples were taken at the days of challenge with DENV-1 (day 308) or DENV-2 (day 252). Post-challenge samples were taken on day 336 for DENV-1 challenge and day 280 for DENV-2 challenge.
§Fold boost in NAb titers after challenge.
